# Quantum prospects for hybrid thin-film lithium niobate on silicon photonics

**DOI:** 10.1007/s12200-022-00006-7

**Published:** 2022-04-11

**Authors:** Jeremy C. Adcock, Yunhong Ding

**Affiliations:** Center for Silicon Photonics for Optical Communication, Technical University of Denmark, 2800 Kgs. Lyngby, Denmark

**Keywords:** Quantum photonics, Quantum information, Quantum communications, Lithium niobate (LN), Silicon photonics

## Abstract

**Abstract:**

Photonics is poised to play a unique role in quantum technology for computation, communications and sensing. Meanwhile, integrated photonic circuits—with their intrinsic phase stability and high-performance, nanoscale components—offer a route to scaling. However, each integrated platform has a unique set of advantages and pitfalls, which can limit their power. So far, the most advanced demonstrations of quantum photonic circuitry has been in silicon photonics. However, thin-film lithium niobate (TFLN) is emerging as a powerful platform with unique capabilities; advances in fabrication have yielded loss metrics competitive with any integrated photonics platform, while its large second-order nonlinearity provides efficient nonlinear processing and ultra-fast modulation. In this short review, we explore the prospects of dynamic quantum circuits—such as multiplexed photon sources and entanglement generation—on hybrid TFLN on silicon (TFLN/Si) photonics and argue that hybrid TFLN/Si photonics may have the capability to deliver the photonic quantum technology of tomorrow.

**Graphical Abstract:**

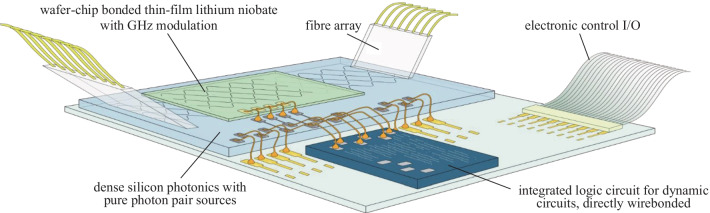

## Introduction

Quantum technology has captured the imagination of scientists and engineers around the world and is poised to play a key role in the blossoming of the information age in the twenty-first century. Today, we are in the era of noisy intermediate scale quantum (NISQ) technology [1], where quantum devices demonstrably outperform traditional computers at some specific tasks [2–4]. Progress in quantum technology is increasingly rapid—the coming decade will reveal both the power of quantum information processing, and the extent of the challenges we face to develop the architectures, nanofabrication techniques, and algorithms needed for large-scale quantum computation and networks.

Photonics remains at the leading edge of quantum technology development, with recent demonstrations of satellite-based quantum communications, loophole-free Bell tests, and demonstrations of quantum advantage [5–10]. Meanwhile, integrated quantum photonics—often seen as the key to scalability—is rapidly advancing in performance and complexity. Nearly all integrated photonics platforms have been used to demonstrate quantum capability [11], with different platforms excelling at different applications. Of particular note is silicon quantum photonics [12, 13], which has seen by far the most activity, across communications [14, 15], computation [16, 17], and sensing [18]. Today, however, progress in silicon quantum photonics is limited by slow or lossy modulation [19].

## Hybrid thin-film lithium niobate on silicon photonics

Thin-film lithium niobate (TFLN) has emerged via recent progress in fabrication [20, 21] as a photonics platform with a unique set of capabilities for high-speed applications [22–24]. While bulk and diffuse-waveguide LN have long been the workhorses of telecommunication technologies, TFLN waveguides offer nanometer-scale confinement, reducing device size, nonlinear thresholds, and switching voltages by orders of magnitude—while commensurately increasing modulation frequencies. With a refractive index of *n* = 2.2, TFLN circuits are reasonably dense, but still require an order of magnitude larger bend radius (~ 100 μm) than silicon circuits (~ 10 μm), which features *n* = 3.5.

Historically, chemical vapor deposition [25], pulsed laser deposition [26], molecular beam epitaxy [27], and RF sputtering [28] have been all be used to fabricate TFLN waveguides. However, none of these is able to achieve high-quality structures in crystalline TFLN. Recently, “smart-cut” technology [26, 29] provided the breakthrough to enable the production of commercial LN on insulator (LNOI) wafers. Instead of growing or depositing LN, the “smart-cut” process consists of first using high-dose ion beams (He^+^ or H^+^) to produce a clean cleaving plane, then wafer bonding to the carrier substrate, thermal annealing to split the original LN substrate along the cleavage plane, and finally polishing for increased smoothness. The “smart-cut” method is able to prepare the single crystalline TFLN on a large area of insulator. Today, wafers up to 6 inch are commercially available from companies such as NanoLN, Partow Technologies, NGK Insulators, and SRICO.

TFLN offers numerous distinct advantages when compared to other platforms for photonic integrated circuits. Modulation speeds of over 100 GHz [30–33] and *V*_π_*L* = 2.8 V·cm [30] have been demonstrated via group-velocity matched electro-optic phase-shifters, leveraging the large χ^(2)^ nonlinearity inherent to LN. Meanwhile, recent advancements in processing have produced ridge waveguides with loss as low as 2.7 dB/m [34]—a critical figure for quantum applications. Waveguide geometry can be used to control the guided mode’s dispersion properties and to engineer nonlinear phenomena. For example, a photonic molecule simulator [35], frequency combs [36], coherent modulation [37], and an integrated spectrometer [38] have all recently been demonstrated. As with bulk LN, periodic inversion of the ferroelectric crystal domain can be used to tailor a TFLN waveguide’s nonlinearity profile [39–42], increasing process efficiencies [40]. This is achieved by applying strong alternating electric fields on a micrometer scale. Here, increased geometrical precision enables more precisely engineered nonlinear profiles, for example, increased purity of nonlinearly generated photon pairs. Efficient thermal modulation has also been shown via introducing thermally isolating voids in the surrounding substrate.

Silicon photonics is a reliable and mature platform for integrated optics [44]. However, simultaneously low-loss and high-speed modulation and switching remains a core challenge. Traditional microheater-based thermal switches suffer from inefficient and slow operation, while high-speed plasma-effect [45] and carrier-depletion based modulators suffer from intrinsic phase-dependent optical loss due to their free carriers [46]. Meanwhile, however, synergy with the information technology sector has bestowed ever greater efficiency for passive components. Examples include − 0.6 dB loss per grating coupler [47–49], − 0.3 dB/cm propagation loss [50, 51] and − 0.1 dB loss per cross-intersection, ideal for quantum applications. Until recently, the spectral purity of photon pairs generated in silicon waveguides was considered a core challenge in silicon quantum photonics. However, recent demonstrations boast 95% purity from ring resonator sources [52], and > 98% from a source based on multimode phase matching [53]. Meanwhile, theory shines a light on improvements beyond 99% [54, 55].

ybrid TFLN/Si photonic devices are constructed by wafer bonding TFLN circuits on top the silicon waveguide layer, spaced with a layer of silicon dioxide of around 100 μm thickness. Recently, scientists have also achieved crystalline silicon thin film and TFLN hybrid wafers by the smart-cut method [26, 29]. This enables efficient coupling between the silicon and TFLN waveguide modes by means of inverted tapers on the silicon and TFLN layers, forming a vertically spaced adiabatic coupler. This adiabatic mode transition can be low-loss (− 0.19 dB) and single-mode [56, 57], though requires precise placement of the LN wafer. High quality, precision etching of TFLN remains a challenge, resulting in grating coupler efficiencies that lag behind other platforms. Today, the state of the art is − 3.5 dB transmission [58–60]. Here hybridization with silicon’s sub-dB grating couplers [47–49] offers a large increase in efficiency.

Few platforms for integrated optics can compete with TFLN’s loss and modulation speed metrics. However, other platforms have specific advantages. For example, platforms based on III-V semiconductors, such as Indium phosphide (InP), gallium arsenide (GaAs), aluminum gallium arsenide (AlGaAs), and their hybrids, offer on-chip optical amplification via their direct bandgap, and therefore allow the integration of single-wavelength lasers. As high refractive index materials, they feature a high-optical confinement and small device footprint, similar to silicon. Meanwhile, integration with silicon devices is also possible via flip-chip bonding and heterogeneous growth and integration techniques [61]. Modulation bandwidths up to 67 GHz (*V*_π_ = 1.5 V) have been shown in InP, approaching the speeds of TFLN [62], though propagation loss metrics lag behind. AlGaAs offers large intrinsic χ^(2)^ and χ^(3)^ nonlinearities, and does not suffer from two-photon absorbtion. While AlGaAs typically suffers from large propagation loss, sidewall passivation techniques have demonstrated losses as low as 1 dB/cm [63]. III-V semiconductor-based Plasmonic modulators can reach speeds over 500 GHz, however the loss associated is too large for quantum applications, typically greater than 5 dB [64]. Integrated graphene [65], meanwhile, may provide a route to ultra-high nonlinearities. Finally, silicon nitride photonics suffers from similar efficiency and speed constraints to silicon, but requires a considerably larger footprint for equivalent circuitry.

## Dynamic quantum photonic circuits

**Fig. 1:**
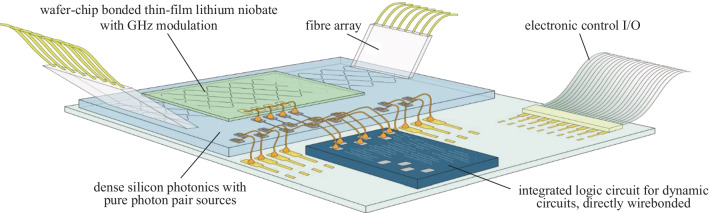
Future TFLN/Si device package designed for dynamic quantum photonic circuits and featuring direct wirebonding for low-latency logic

Dynamic quantum photonic circuitry (DQPC)—involving quantum measurement and feed-forward drive information processing—are vital to large-scale quantum photonic technologies, and require fast, low loss photonic circuitry, as well as low-latency detection-logic and fast switching, as shown in Fig. 1. These same requirements enable applications across quantum photonic technologies. Applied at scale, DQPC yields linear optical quantum computation (LOQC), continuous variables (CVs) quantum computing [66–69], as well as unlimited-distance quantum communications via quantum repeaters [70]. In these schemes, quantum protocols (algorithms, communications channels, etc.) are implemented via local adaptive measurements on large entangled resource states. These lattice-like quantum states, whose entanglement structure (represented as a graph) provide error or loss tolerance to their protocol when measured with the correct adaptive algorithm. In many architectures these resource states are themselves built using DQPC.

### Photon source multiplexing

Photon source multiplexing is one of the elementary examples of a dynamic circuit. Silicon’s spontaneous four-wave mixing (SFWM) photon pair sources are probabilistic; they rely on the postselection of photon pairs from a squeezed vacuum state with a probability necessarily *p* < 0.1. This results in exponentially poor scaling of success for multiple sources, and places limitations on resource state generation [71]. Photon source multiplexing, however, offers a clear path to determinism, whereby fast, low-loss switches dynamically route a single photons from many multiplexed photon pair sources to one output. These sources can be separated by any photonic degree of freedom, for example, space, time, or frequency [72–77].

Optical loss strongly suppresses advantage in multiplexing schemes, and only recently have significant enhancements of photon probability been measured. Here, a low-loss bulk-optical system multiplexed up to 40 time bins, culminating in a photon output probability of 67% with the second-order correlation function *g*^(2)^(0) = 0.28, indicating considerable two-photon component [75]. So far, however, integrated examples are limited to off-chip switching [76, 77]. In contrast, solid-state emitters, such as quantum dots, have demonstrated up to 57% output probability to optical fiber [78, 79]. However, the best examples of quantum dot based sources emit photons with wavelengths around 800 nm, far from the vital telecommunications O- and C-bands, and require bulky and expensive cryogenic operation. Meanwhile, DQPC—and therefore high-speed and low loss modulation—is required for measurement-based quantum protocols, no matter where the photons come from. To complete the stack, heralded entangling gates can be multiplexed using precisely the same techniques and technology in order to generate large entangled resource states for error- and/or loss-tolerant quantum protocols [80, 81, 67].

Using state of the art loss metrics (see Table 1), a relatively modest repetition rate of 10 GHz implies the optical transmission of a temporal storage loop to be as low as − 0.05 dB per round trip. Meanwhile SFWM based photon pair sources have been demonstrated in silicon with repetition rates of up to 10 GHz [82]. A simple model [75] shows that multiplexing based on a silicon waveguide source and single temporal loop in the TFLN layer could produce a photon with probability around 60%. With the output clocked to the recovery time of a single superconducting nanowire single photon detector (around 50 ns recovery for commercial devices) an on-chip single photon flux of 12 MHz is achieved, which is comparable with state-of-the-art solid-state sources before coupling to the application (e.g., integrated photonics). Reducing detector deadtime to 1 ns [83] could yield an on-chip rate of 300 MHz. Spatial multiplexed sources, requiring *n* detectors for *n* multiplexed sources, show even more promising results, at the cost of additional resources and device footprint. Heralded entangling gates, such as type-I and type-II fusion have success probabilities greater than 50% (c.f., less than 10% for photon pair generation), and so are less technically demanding to multiplex than sources.

**Table 1:** State-of-the-art component metrics in TFLN and silicon photonics

Device/component	Figure of merit	References
TFLN modulator bandwidth	110 GHz	[30]
TFLN modulator loss	− 0.5 dB	[31]
TFLN modulator *V*_π_*L*	2.8 V·cm	[31]
Si/TFLN mode converter loss	− 0.19 dB	[33]
TFLN propagation loss	− 2.7 dB/m	[34]
Si propagation loss	− 2.7 dB/m	[50, 51]
TFLN side coupler	− 1.32 dB (TE)	[86]
TFLN grating coupler	− 3.5 dB (TE)	[58, 59]
Si grating coupler	− 0.36 dB (1200 nm), − 0.5 dB (1550 nm)	[48, 49]

To increase the efficiency in DQPC, and move toward quantum photonic systems-on-a-chip, the detect-switch latency must be reduced. Current implementations based on field programmable gate array logic demonstrate latencies of around 100 ns, or around 20 m of photon storage in optical fiber, which is infeasible on chip. To improve this, devices could be placed inside a cryostat with single photon detectors, which is enabled by the minute power dissipation of TFLN’s electro-optic modulation technology. Furthermore, direct wire-bonding of the photonic integrated circuit to a dedicated electronic logic chip [84] could yield latencies of around 1 ns. Eventually, electronic/optical co-integration in silicon may reduce this yet farther.

## Concluding remarks

Hybrid TFLN/Si offers dense, high-performance optics combined with low loss and ultra-fast modulation, and will undoubtedly play a key role in the development of photonic information processing—both quantum and classical—as the technology becomes available to end users. Other platforms, such as Si/BTO [85], and strained silicon show similar potential, though speed and loss and speed metrics lag behind those of TFLN, and demonstrations so far have relied on specialized silicon wafer production, rather than versatile wafer bonding techniques. In the coming years, quantum photonics will continue to benefit from parallel development of integrated photonics and electronics, driven by society’s demand for bandwidth and connectivity, while quantum-specialized processes will provide the state-of-the-art as we progress through the NISQ era of quantum information processing.

## References

[1] Preskill J (2018). Quantum computing in the NISQ era and beyond. Quantum.

[2] Arute F, Arya K, Babbush R, Bacon D, Bardin JC, Barends R, Biswas R, Boixo S, Brandao FGSL, Buell DA, Burkett B, Chen Y, Chen Z, Chiaro B, Collins R, Courtney W, Dunsworth A, Farhi E, Foxen B, Fowler A, Gidney C, Giustina M, Graff R, Guerin K, Habegger S, Harrigan MP, Hartmann MJ, Ho A, Hoffmann M, Huang T, Humble TS, Isakov SV, Jeffrey E, Jiang Z, Kafri D, Kechedzhi K, Kelly J, Klimov PV, Knysh S, Korotkov A, Kostritsa F, Landhuis D, Lindmark M, Lucero E, Lyakh D, Mandrà S, McClean JR, McEwen M, Megrant A, Mi X, Michielsen K, Mohseni M, Mutus J, Naaman O, Neeley M, Neill C, Niu MY, Ostby E, Petukhov A, Platt JC, Quintana C, Rieffel EG, Roushan P, Rubin NC, Sank D, Satzinger KJ, Smelyanskiy V, Sung KJ, Trevithick MD, Vainsencher A, Villalonga B, White T, Yao ZJ, Yeh P, Zalcman A, Neven H, Martinis JM (2019). Quantum supremacy using a programmable superconducting processor. Nature.

[3] Zhong, H.S., Deng, Y.H., Qin, J., Wang, H., Chen, M.C., Peng, L.C., Luo, Y.H., Wu, D., Gong, S.Q., Su, H., Hu, Y.: Phase-programmable gaussian boson sampling using stimulated squeezed light. arxiv preprint arxiv:2106.15534 (2021) 10.1103/PhysRevLett.127.180502 34767431

[4] Wu YL, Bao WS, Cao SR, Chen FS, Chen MC, Chen XW, Chung TS, Deng H, Du YJ, Fan DJ, Gong M, Guo C, Guo C, Guo SJ, Han LC, Hong LY, Huang HL, Huo YH, Li LP, Li N, Li SW, Li Y, Liang FT, Lin C, Lin J, Qian HR, Qiao D, Rong H, Su H, Sun LH, Wang LY, Wang SY, Wu DC, Xu Y, Yan K, Yang WF, Yang Y, Ye YS, Yin JH, Ying C, Yu JL, Zha C, Zhang C, Zhang HB, Zhang KL, Zhang YM, Zhao H, Zhao YW, Zhou L, Zhu QL, Lu CY, Peng CZ, Zhu XB, Pan JW (2021). Strong quantum computational advantage using a superconducting quantum processor. Phys. Rev. Lett..

[5] Liao SK, Cai WQ, Liu WY, Zhang L, Li Y, Ren JG, Yin J, Shen Q, Cao Y, Li ZP, Li FZ, Chen XW, Sun LH, Jia JJ, Wu JC, Jiang XJ, Wang JF, Huang YM, Wang Q, Zhou YL, Deng L, Xi T, Ma L, Hu T, Zhang Q, Chen YA, Liu NL, Wang XB, Zhu ZC, Lu CY, Shu R, Peng CZ, Wang JY, Pan JW (2017). Satellite-to-ground quantum key distribution. Nature.

[6] Ren JG, Xu P, Yong HL, Zhang L, Liao SK, Yin J, Liu WY, Cai WQ, Yang M, Li L, Yang KX, Han X, Yao YQ, Li J, Wu HY, Wan S, Liu L, Liu DQ, Kuang YW, He ZP, Shang P, Guo C, Zheng RH, Tian K, Zhu ZC, Liu NL, Lu CY, Shu R, Chen YA, Peng CZ, Wang JY, Pan JW (2017). Ground-to-satellite quantum teleportation. Nature.

[7] Giustina M, Versteegh MAM, Wengerowsky S, Handsteiner J, Hochrainer A, Phelan K, Steinlechner F, Kofler J, Larsson J, Abellán C, Amaya W, Pruneri V, Mitchell MW, Beyer J, Gerrits T, Lita AE, Shalm LK, Nam SW, Scheidl T, Ursin R, Wittmann B, Zeilinger A (2015). Significant-loophole-free test of Bell’s theorem with entangled photons. Phys. Rev. Lett..

[8] Hensen B, Bernien H, Dréau AE, Reiserer A, Kalb N, Blok MS, Ruitenberg J, Vermeulen RF, Schouten RN, Abellán C, Amaya W, Pruneri V, Mitchell MW, Markham M, Twitchen DJ, Elkouss D, Wehner S, Taminiau TH, Hanson R (2015). Loophole-free Bell inequality violation using electron spins separated by 1.3 kilometres. Nature.

[9] Shalm LK (2015). Strong loophole-free test of local realism. Phys. Rev. Lett..

[10] Bromley TR (2020). Applications of near-term photonic quantum computers: software and algorithms. Quan. Sci. Technol..

[11] Wang J, Sciarrino F, Laing A, Thompson MG (2020). Integrated photonic quantum technologies. Nat. Photon..

[12] Silverstone, J.W., Wang, J., Bonneau, D., Sibson, P., Santagati, R., Erven, C., O'Brien, J.L., Thompson, M.G.: Silicon quantum photonics. In: Proceedings of International Conference on Optical MEMS and Nanophotonics (OMN). Singapore: IEEE (2016)

[13] Adcock JC, Bao J, Chi Y, Chen X, Bacco D, Gong Q, Oxenlowe LK, Wang J, Ding Y (2021). Advances in silicon quantum photonics. IEEE J. Sel. Top. Quantum Electron..

[14] Sibson P, Kennard JE, Stanisic S, Erven C, O’Brien JL, Thompson MG (2017). Integrated silicon photonics for high-speed quantum key distribution. Optica.

[15] Llewellyn D, Ding Y, Faruque II, Paesani S, Bacco D, Santagati R, Qian YJ, Li Y, Xiao YF, Huber M, Malik M, Sinclair GF, Zhou X, Rottwitt K, O’Brien JL, Rarity JG, Gong Q, Oxenlowe LK, Wang J, Thompson MG (2020). Chip-to-chip quantum teleportation and multi-photon entanglement in silicon. Nat. Phys..

[16] Vigliar C, Paesani S, Ding YH, Adcock JC, Wang JW, Morley-Short S, Bacco D, Oxenløwe LK, Thompson MG, Rarity JG, Laing A (2020). Error protected qubits in a silicon photonic chip. Nature Phys..

[17] Paesani S, Ding Y, Santagati R, Chakhmakhchyan L, Vigliar C, Rottwitt K, Oxenløwe LK, Wang J, Thompson MG, Laing A (2019). Generation and sampling of quantum states of light in a silicon chip. Nat. Phys..

[18] Ono T, Sinclair GF, Bonneau D, Thompson MG, Matthews JCF, Rarity JG (2019). Observation of nonlinear interference on a silicon photonic chip. Opt. Lett..

[19] Rudolph T (2017). Why I am optimistic about the silicon-photonic route to quantum computing. APL Photon..

[20] Levy M, Osgood RM, Liu R, Cross LE, Cargill GSIII, Kumar A, Bakhru H (1998). Fabrication of single-crystal lithium niobate films by crystal ion slicing. Appl. Phys. Lett..

[21] Rabiei P, Ma J, Khan S, Chiles J, Fathpour S (2013). Heterogeneous lithium niobate photonics on silicon substrates. Opt. Express.

[22] Poberaj G, Hu H, Sohler W, Günter P (2012). Lithium niobate on insulator (LNOI) for micro-photonic devices. Laser Photonics Rev..

[23] Bazzan M, Sada C (2015). Optical waveguides in lithium niobate: recent developments and applications. Appl. Phys. Rev..

[24] Weigel PO, Zhao J, Fang K, Al-Rubaye H, Trotter D, Hood D, Mudrick J, Dallo C, Pomerene AT, Starbuck AL, DeRose CT, Lentine AL, Rebeiz G, Mookherjea S (2018). Bonded thin film lithium niobate modulator on a silicon photonics platform exceeding 100 GHz 3-dB electrical modulation bandwidth. Opt. Express.

[25] Sakashita Y, Segawa H (1995). Preparation and characterization of LiNbO3 thin films produced by chemical-vapor deposition. J. Appl. Phys..

[26] Nakata Y, Gunji S, Okada T, Maeda M (2004). Fabrication of LiNbO3 thin films by pulsed laser deposition and investigation of nonlinear properties. Appl. Phys. A.

[27] Gitmans F, Sitar Z, Günter P (1995). Growth of tantalum oxide and lithium tantalate thin films by molecular beam epitaxy. Vacuum.

[28] Lansiaux X, Dogheche E, Remiens D, Guilloux-viry M, Perrin A, Ruterana P (2001). LiNbO3 thick films grown on sapphire by using a multistep sputtering process. J. Appl. Phys..

[29] Bruel M (1995). Silicon on insulator material technology. Electron. Lett..

[30] Mercante AJ, Yao P, Shi S, Schneider G, Murakowski J, Prather DW (2016). 110 GHz CMOS compatible thin film LiNbO_3_ modulator on silicon. Opt. Express.

[31] Wang C, Zhang M, Chen X, Bertrand M, Shams-Ansari A, Chandrasekhar S, Winzer P, Lončar M (2018). Integrated lithium niobate electro-optic modulators operating at CMOS-compatible voltages. Nature.

[32] Wang C, Zhang M, Stern B, Lipson M, Lončar M (2018). Nanophotonic lithium niobate electro-optic modulators. Opt. Express.

[33] He M, Xu M, Ren Y, Jian J, Ruan Z, Xu Y, Gao S, Sun S, Wen X, Zhou L, Liu L, Guo C, Chen H, Yu S, Liu L, Cai X (2019). High-performance hybrid silicon and lithium niobate Mach-Zehnder modulators for 100 Gbit s−1 and beyond. Nat. Photonics.

[34] Zhang M, Wang C, Cheng R, Shams-Ansari A, Lončar M (2017). Monolithic ultra-high-Q lithium niobate microring resonator. Optica.

[35] Zhang M, Wang C, Hu Y, Shams-Ansari A, Ren T, Fan S, Lončar M (2019). Electronically programmable photonic molecule. Nat. Photonics.

[36] Wang C, Zhang M, Yu M, Zhu R, Hu H, Loncar M (2019). Monolithic lithium niobate photonic circuits for Kerr frequency comb generation and modulation. Nat. Commun..

[37] Xu M, He M, Zhang H, Jian J, Pan Y, Liu X, Chen L, Meng X, Chen H, Li Z, Xiao X, Yu S, Yu S, Cai X (2020). High-performance coherent optical modulators based on thin-film lithium niobate platform. Nat. Commun..

[38] Pohl D, Reig Escalé M, Madi M, Kaufmann F, Brotzer P, Sergeyev A, Guldimann B, Giaccari P, Alberti E, Meier U, Grange R (2020). An integrated broadband spectrometer on thin-film lithium niobate. Nat. Photonics.

[39] Sun D, Zhang Y, Wang D, Song W, Liu X, Pang J, Geng D, Sang Y, Liu H (2020). Microstructure and domain engineering of lithium niobate crystal films for integrated photonic applications. Light Sci. Appl..

[40] Li H, Ma B (2020). Research development on fabrication and optical properties of nonlinear photonic crystals. Front. Optoelectron..

[41] Chen F (2019). Laser-written three dimensional nonlinear photonic crystals. Front. Optoelectron..

[42] Lu J, Surya JB, Liu X, Bruch AW, Gong Z, Xu Y, Tang HX (2019). Periodically poled thin-film lithium niobate microring resonators with a second-harmonic generation efficiency of 250000%/W. Optica.

[43] Liu X, Ying P, Zhong X, Xu J, Han Y, Yu S, Cai X (2020). Highly efficient thermo-optic tunable micro-ring resonator based on an LNOI platform. Opt. Lett..

[44] Thomson D (2016). Roadmap on silicon photonics. J. Opt..

[45] Treyz GV, May PG, Halbout JM (1991). Silicon Mach-Zehnder waveguide interferometers based on the plasma dispersion effect. Appl. Phys. Lett..

[46] Liu A, Liao L, Rubin D, Nguyen H, Ciftcioglu B, Chetrit Y, Izhaky N, Paniccia M (2007). High-speed optical modulation based on carrier depletion in a silicon waveguide. Opt. Express.

[47] Ding Y, Peucheret C, Ou H, Yvind K (2014). Fully etched apodized grating coupler on the SOI platform with −0.58 dB coupling efficiency. Opt. Lett..

[48] Notaros, J., Pavanello, F., Wade, M. T., Gentry, C. M., Atabaki, A., Alloatti, L., Ram, R. J., Miloš, A. P. Ultra-efficient CMOS fiber-to-chip grating couplers. In: Proceedings of Optical Fiber Communications Conference and Exhibition (OFC). Anaheim: IEEE (2016)

[49] Hoppe N, Zaoui WS, Rathgeber L, Wang Y, Klenk RH, Vogel W, Kaschel M, Portalupi SL, Burghartz J, Berroth M (2020). Ultra-efficient silicon-on-insulator grating couplers with backside metal mirrors. IEEE J. Sel. Top. Quantum Electron..

[50] Horikawa T, Shimura D, Mogami T (2016). Low-loss silicon wire waveguides for optical integrated circuits. MRS Commun..

[51] Biberman A, Shaw MJ, Timurdogan E, Wright JB, Watts MR (2012). Ultralow-loss silicon ring resonators. Opt. Lett..

[52] Liu Y, Wu C, Gu X, Kong Y, Yu X, Ge R, Cai X, Qiang X, Wu J, Yang X, Xu P (2020). High-spectral-purity photon generation from a dual-interferometer-coupled silicon microring. Opt. Lett..

[53] Paesani S, Borghi M, Signorini S, Maïnos A, Pavesi L, Laing A (2020). Near-ideal spontaneous photon sources in silicon quantum photonics. Nat. Commun..

[54] Christensen, J.B., Koefoed, J.G., Rottwitt, K., McKinstrie, C.: Engineering spectrally unentangled photon pairs from nonlinear microring resonators through pump manipulation. arxiv preprint arxiv:1711.02401 (2017) 10.1364/OL.43.000859 29444012

[55] Vernon Z, Menotti M, Tison CC, Steidle JA, Fanto ML, Thomas PM, Preble SF, Smith AM, Alsing PM, Liscidini M, Sipe JE (2017). Truly unentangled photon pairs without spectral filtering. Opt. Lett..

[56] Zhu HB (2021). Hybrid mono-crystalline silicon and lithium niobate thin films. Chin. Opt. Lett..

[57] Weigel PO, Savanier M, DeRose CT, Pomerene AT, Starbuck AL, Lentine AL, Stenger V, Mookherjea S (2016). Lightwave circuits in lithium niobate through hybrid waveguides with silicon photonics. Sci. Rep..

[58] Krasnokutska I, Chapman RJ, Tambasco JJ, Peruzzo A (2019). High coupling efficiency grating couplers on lithium niobate on insulator. Opt. Express.

[59] Chen B, Ruan Z, Hu J, Wang J, Lu C, Lau APT, Guo C, Chen K, Chen P, Liu L (2021). Two-dimensional grating coupler on an X-cut lithium niobate thin-film. Opt. Express.

[60] Ruan Z, Hu J, Xue Y, Liu J, Chen B, Wang J, Chen K, Chen P, Liu L (2020). Metal based grating coupler on a thin-film lithium niobate waveguide. Opt. Express.

[61] Bowers, J. E., Liu, A. Y. A comparison of four approaches to photonic integration. In: Proceedings of Optical Fiber Communication Conference. Los Angeles: IEEE (2017)

[62] Ogiso Y, Ozaki J, Ueda Y, Kashio N, Kikuchi N, Yamada E, Tanobe H, Kanazawa S, Yamazaki H, Ohiso Y, Fujii T, Kohtoku M (2017). Over 67 GHz bandwidth and 15 V Vπ InP-based optical IQ modulator with nipn heterostructure. J. Lightwave Technol..

[63] Ottaviano L, Pu M, Semenova E, Yvind K (2016). Low-loss high-confinement waveguides and microring resonators in AlGaAs-on-insulator. Opt. Lett..

[64] Burla M (2019). 500 GHz plasmonic Mach-Zehnder modulator enabling sub-THz microwave photonics. APL Photon..

[65] Zhong C, Li J, Lin H (2020). Graphene-based all-optical modulators. Front. Optoelectron..

[66] Knill E, Laflamme R, Milburn GJ (2001). A scheme for efficient quantum computation with linear optics. Nature.

[67] Bartolucci, S., Birchall, P., Bombin, H., Cable, H., Dawson, C., Gimeno-Segovia, M., Johnston, E., Kieling, K., Nickerson, N., Pant, M., Pastawski, F., Rudolph, T., Sparrow, C.: Fusion-based quantum computation. arxiv preprint arxiv:2101.09310 (2021) 10.1038/s41467-023-36493-1 PMC9938229 36805650

[68] Takeda S, Furusawa A (2019). Toward large-scale fault-tolerant universal photonic quantum computing. APL Photon..

[69] Bourassa JE, Alexander RN, Vasmer M, Patil A, Tzitrin I, Matsuura T, Su D, Baragiola BQ, Guha S, Dauphinais G, Sabapathy KK, Menicucci NC, Dhand I (2021). Blueprint for a scalable photonic fault-tolerant quantum computer. Quantum.

[70] Azuma K, Tamaki K, Lo HK (2015). All-photonic quantum repeaters. Nat. Commun..

[71] Adcock JC (2018). Hard limits on the postselectability of optical graph states. Quan. Sci. Technol..

[72] Migdall AL, Branning D, Castelletto S (2002). Tailoring single-photon and multiphoton probabilities of a single-photon on-demand source. Phys. Rev. A.

[73] Pittman TB, Jacobs BC, Franson P (2002). Single photons on pseudodemand from stored parametric down-conversion. Phys. Rev. A.

[74] Bonneau D (2015). Effect of loss on multiplexed single-photon sources. N. J. Phys..

[75] Fumihiro K, Kwiat P (2019). High-efficiency single-photon generation via large-scale active time multiplexing. Sci. Adv..

[76] Collins MJ, Xiong C, Rey IH, Vo TD, He J, Shahnia S, Reardon C, Krauss TF, Steel MJ, Clark AS, Eggleton BJ (2013). Integrated spatial multiplexing of heralded single-photon sources. Nat. Commun..

[77] Joshi C, Farsi A, Clemmen S, Ramelow S, Gaeta AL (2018). Frequency multiplexing for quasi-deterministic heralded single-photon sources. Nat. Commun..

[78] Thomas S, Billard M, Coste N, Wein S, Ollivier P, Krebs O, Tazaïrt L, Harouri A, Lemaitre A, Sagnes I, Anton C, Lanco L, Somaschi N, Loredo J, Senellart P (2021). Bright polarized single-photon source based on a linear dipole. Phys. Rev. Lett..

[79] Tomm N, Javadi A, Antoniadis NO, Najer D, Löbl MC, Korsch AR, Schott R, Valentin SR, Wieck AD, Ludwig A, Warburton RJ (2021). A bright and fast source of coherent single photons. Nat. Nanotechnol..

[80] Bartolucci, S., Birchall, P., Gimeno-Segovia, M., Johnston, E., Kieling, K., Mihir Pant, M., Rudolph, T., Smith, J., Sparrow, C., Vidrighin, M.: Creation of entangled photonic states using linear optics. arxiv preprint arxiv:2106.13825 (2021)

[81] Paesani S, Bulmer J, Jones A, Santagati R, Laing A (2021). Scheme for universal high-dimensional quantum computation with linear optics. Phys. Rev. Lett..

[82] Zhang X, Bell B, Pelusi M, He J, Geng W, Kong Y, Zhang P, Xiong C, Eggleton BJ (2017). High repetition rate correlated photon pair generation in integrated silicon nanowires. Appl. Opt..

[83] Münzberg J, Vetter A, Beutel F, Hartmann W, Ferrari S, Pernice WHP, Rockstuhl C (2018). Superconducting nanowire single-photon detector implemented in a 2D photonic crystal cavity. Optica.

[84] Tasker J F, Frazer J, Ferranti G, Allen F, Brunel L, Tanzilli S, D'Auria V, Matthews J. 9~GHz measurement of squeezed light by interfacing silicon photonics and integrated electronics. arxiv preprint arxiv:2009.14318 (2020)

[85] Eltes F, Villarreal-Garcia GE, Caimi D, Siegwart H, Gentile AA, Hart A, Stark P, Marshall GD, Thompson MG, Barreto J, Fompeyrine J, Abel S (2020). An integrated optical modulator operating at cryogenic temperatures. Nat. Mater..

[86] Li Y, Lan T, Li J, Wang Z (2020). High-efficiency edge-coupling based on lithium niobate on an insulator wire waveguide. Appl. Opt..

